# Imaging-Based Spatial Transcriptomics: Data Interpretation Methods and Biomedical Applications

**DOI:** 10.3390/biology15120900

**Published:** 2026-06-08

**Authors:** Wenhao Li, Yuan Zhou

**Affiliations:** Department of Biomedical Informatics, State Key Laboratory of Vascular Homeostasis and Remodeling, School of Basic Medical Sciences, Peking University, Beijing 100191, China; 1910305223@pku.edu.cn

**Keywords:** imaging-based spatial transcriptomics, barcode decoding, cell segmentation, cell typing, spatial transcriptome data interpretation

## Abstract

Imaging-based spatial transcriptomics has advanced from low-plex single-molecule fluorescence in situ hybridization to a diverse set of highly multiplexed platforms, with recent multimodal and pathology-compatible capabilities. Despite major differences in chemistry, coding, and imaging strategies across different platforms, their biological interpretation often converges on a few notable computational biology problems. This review examines imaging-based spatial transcriptomics through the lens of data interpretation and applications, focusing on the analytical framework that converts raw fluorescence signals or accompanying in situ sequencing data into molecule-, cell-, and tissue-level representations. We discuss the key challenges in preprocessing, registration, restoration, feature detection, barcode decoding, molecule calling, cell segmentation, transcript assignment, probabilistic cell typing, spatial-domain inference, and atlas integration. We also highlight how optical crowding, tissue thickness, panel bias, and multimodal complexity increase computational difficulty. Finally, we summarize applications of imaging-based spatial transcriptomics techniques, ranging from subcellular RNA localization to atlas-scale and pathology-aware spatial analysis.

## Simple Summary

Spatial transcriptomics allows researchers to measure gene expression while preserving the positions of cells and molecules within tissues. Imaging-based spatial transcriptomics methods do this by directly visualizing RNA molecules or amplified signals in tissue samples, often at single-cell or subcellular resolution. However, these assays generate complex microscopy images, and reliable biological interpretation depends on careful computational processing. This review aims to help readers understand how these data can be interpreted reliably. It summarizes the development of imaging-based spatial transcriptomics and explains how raw image signals are transformed into molecular maps, cell-level measurements, tissue region annotations, and biological insights. It highlights key computational analytical steps and major challenges such as signal crowding, barriers in thick tissues, data integration, and error propagation issues. Biomedical applications ranging from subcellular RNA localization to tissue atlases and pathology-compatible disease studies are also discussed.

## 1. Introduction

Gene expression profiling has drastically accelerated biomedical studies and clinical applications by linking development, physiology and diseases to a wide spectrum of molecular features. However, traditional bulk RNA sequencing averages signals across large cell populations and therefore obscures the cellular heterogeneity that defines most tissues [1]. Single-cell and single-nucleus transcriptomic technologies were developed to address this limitation and have greatly improved the characterization of cell types, transient states, and lineage relationships [2]. Yet these dissociative approaches disrupt tissue architecture, remove tissue morphology, and erase the spatial relationships among cells and transcripts [3,4].

**Figure 1:**
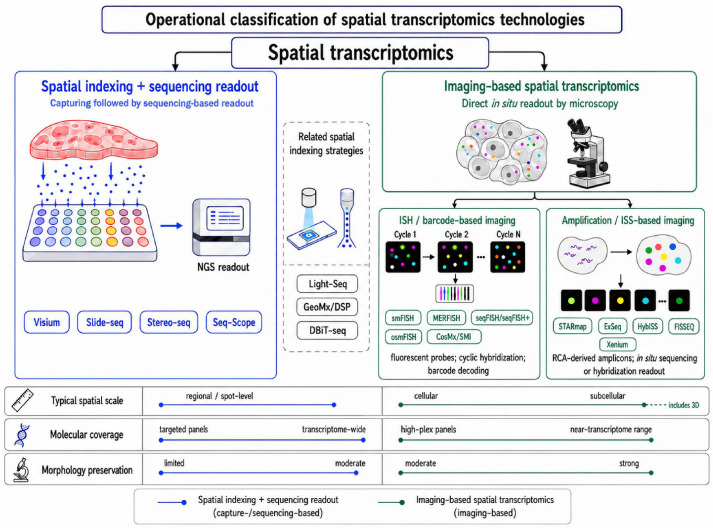
Operational classification of spatial transcriptomics technologies. Spatial transcriptomic methods are grouped into capture-based approaches (spatial indexing) and imaging-based approaches, with the latter including ISH/barcode-based and amplification-based or in situ sequencing-based methods. The spatial scale, molecular coverage and morphology preservation are roughly compared, where blue bars indicate capture-based techniques and green bars indicate imaging-based approaches.

To this end, spatial transcriptomics has emerged to overcome this limitation by preserving positional information while measuring gene expression. Spatial transcriptomic technologies are commonly grouped into two broad categories: spatial-indexing or capture-based approaches, in which captured RNA molecules or molecular tags from each spatial spot are read out by sequencing; and imaging-based approaches, in which spatial expression patterns are directly read out by microscopy [5,6,7,8] (Figure 1). Within the imaging-based family, hybridization-based methods derived from in situ hybridization (ISH) detect RNA molecules through fluorescence signals, whereas in situ sequencing (ISS)-related or amplification-based methods detect amplified products through cyclic imaging, sequencing-by-ligation, sequencing-by-hybridization, or barcode decoding. Thus, although the term “in situ sequencing” is used differently across methods, these approaches are considered here as part of imaging-based spatial transcriptomics because molecular identities are ultimately recovered from spatially resolved microscopic images rather than from dissociated sequencing libraries [5,6,7,8]. In this review, we focus on imaging-based spatial transcriptomics, including both ISH-based methods and ISS-based (or amplification-based) methods. Imaging-based approaches occupy a distinctive position because they directly detect RNA molecules or derived amplification products in situ, therefore often achieving single-cell or subcellular resolution [4,5]. Starting from single-molecule fluorescence in situ hybridization (smFISH), the field has expanded into a diverse set of highly multiplexed platforms, including multiplexed error-robust fluorescence in situ hybridization (MERFISH), sequential fluorescence in situ hybridization (seqFISH) and its high-plex extension seqFISH+, ouroboros single-molecule fluorescence in situ hybridization (osmFISH), spatially resolved transcript amplicon readout mapping (STARmap), and expansion sequencing (ExSeq) [5,9,10,11,12]. Newer hybridization-based and padlock/rolling circle amplification (RCA)-mediated approaches, including hybridization-based in situ sequencing (HybISS), 10x Genomics Xenium, reverse-padlock amplicon-encoding fluorescence in situ hybridization (RAEFISH), and profiling of RNA in situ through single-round imaging (PRISM), further expand this methodological family [13,14,15,16]. Despite major differences in chemistry, coding strategies, and sample compatibility, these methods share a common analytical challenge: raw fluorescence or in situ sequencing-amplified signals must be converted into reliable molecular, cellular, and tissue-level representations and interpretations [2,9].

Accordingly, computation is not a downstream accessory in imaging-based spatial transcriptomics but a central determinant of data quality and biological interpretation [17,18,19]. The results of a spatial transcriptomics assay often depend on preprocessing, registration, denoising, feature detection, barcode decoding, molecule calling, cell segmentation, transcript assignment, cell typing, and atlas-scale integration. These tasks become especially difficult in dense tissues, three-dimensional samples, and multimodal workflows, where signal overlap, boundary uncertainty, and reference dependence can strongly bias downstream inference [17,18,19,20].

This review focuses on imaging-based spatial transcriptomics from the perspective of data interpretation, with particular emphasis on how technological evolution reshapes computational problems, how analytical pipelines transform images into interpretable spatial measurements, and how these advances enable applications ranging from subcellular RNA localization to atlas-scale and pathology-aware spatial analysis [4,19,20].

## 2. Technological Evolution of Imaging-Based Spatial Transcriptomics

### 2.1. From smFISH to Combinatorial Barcoding and Error-Robust Decoding

Imaging-based spatial transcriptomics has emerged from single-molecule fluorescence in situ hybridization (smFISH), which enables individual RNA molecules to be visualized as diffraction-limited spots in fixed cells using multiple probes targeting the same transcript [21,22]. Compared with conventional in situ hybridization, smFISH provides an amplification-free and quantitative framework for in situ RNA counting while preserving subcellular positional information. However, the multiplexing capacity of smFISH is limited and cannot fulfill the current requirement of spatial transcriptomics, as only a small number of transcripts can be resolved simultaneously with conventional fluorescence channels [5,23].

One primary solution is sequential hybridization, in which transcripts fixed in one place are identified not by a single fluorophore in one imaging round but by an ordered fluorescence barcode read across repeated cycles of hybridization, imaging, and probe stripping [23]. This strategy transforms transcript identification from one-round spectral separation into a combinatorial coding problem across imaging rounds, with a theoretical coding capacity that scales as *F^N^*, where *F* is the number of fluorophores and *N* is the number of hybridization rounds [23]. Conceptually, this framework is the basis for later highly multiplexed imaging strategies built on multi-round barcode readouts.

MERFISH advances this logic by incorporating error-robust barcode design through codebooks with constrained Hamming distance, thereby making transcript identity recovery an explicit decoding problem under noisy measurements [5]. Here, MERFISH is used as a representative example of error-robust hybridization-based imaging. In this method, multiple probes bind to each RNA molecule and carry short DNA readout sequences that are interrogated across successive imaging rounds. By recording which signals appear in each round, the identity of each RNA is reconstructed, while the use of multiple probes per target helps maintain strong detection sensitivity. Beyond MERFISH, imaging-based spatial transcriptomic methods mitigate errors through different design principles, including Hamming-distance-based redundancy in MERFISH, pseudocolor-based density dilution and shorter barcodes in seqFISH+, and signal amplification followed by sequencing- or hybridization-based readout in amplification-assisted methods such as STARmap and ExSeq [5,9,11,12]. Recent codebook-optimization work further illustrates that multiplexing capacity can be improved computationally, but such gains must be balanced against imaging time, probe density, and molecular crowding [24].

### 2.2. Optical Crowding and Distinct Strategies for Scaling Multiplexed Imaging

**Figure 2:**
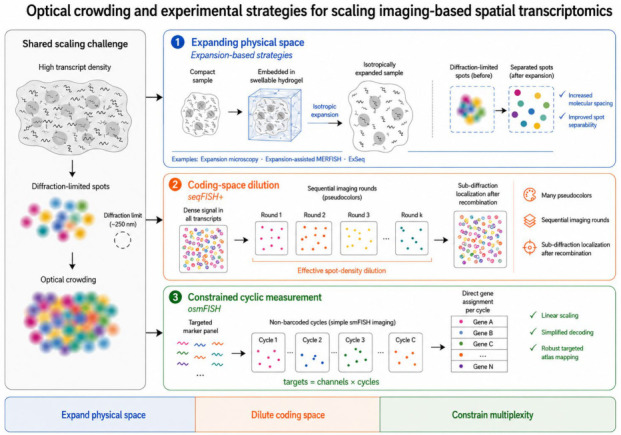
Optical crowding and experimental strategies for scaling imaging-based spatial transcriptomics. Increasing transcript density causes overlap between diffraction-limited RNA signals. This bottleneck can be mitigated by expanding physical space, diluting coding space, or constraining multiplexity through targeted cyclic measurements.

As imaging-based spatial transcriptomic methods have expanded toward larger gene panels and higher-abundance transcripts, optical crowding has emerged as a major constraint on further scaling [5,9,25]. Because each RNA molecule is detected as a diffraction-limited fluorescent spot, increasing molecular density leads to fluorescence signal overlap and reduces the accuracy of localization and downstream decoding [5,9,25]. This limit is ultimately constrained by the point-spread function of the microscope: the lateral resolution can be approximated as $d_{xy}\approx 0.61\lambda /NA$, whereas the axial resolution scales approximately as $d_{z}\approx 2n\lambda /NA^{2}$, where λ is the effective imaging wavelength, *NA* is the numerical aperture, and *n* is the refractive index of the immersion medium [26]. Thus, within a 3D tissue volume, the maximum density of individually detectable RNA molecules is limited by the effective point-spread-function volume, and overlapping emitters within this volume become difficult to localize or decode independently [9,25,26]. In multicycle assays, this physical density limit is compounded by signal instability: fluorophore photobleaching, incomplete probe removal, and cycle-dependent signal decay can introduce missing bits or uneven readout intensities, thereby increasing barcode dropout and decoding ambiguity as the number of hybridization rounds increases [5,9]. Therefore, a major part of subsequent methodological development in highly multiplexed imaging-based spatial transcriptomics can be understood as efforts to reduce, redistribute, or circumvent local signal density while maintaining barcode readability across repeated imaging cycles [5,9,10,25]. These strategies followed three different scaling approaches: expanding physical space [25], diluting coding space [9], or constraining multiplexity to preserve robustness [10] (Figure 2).

One strategy is to physically increase the spacing between RNA molecules before optical readout. Expansion fluorescence in situ hybridization (ExFISH) showed that transcripts could be covalently anchored within a swellable polymer network and visualized after isotropic tissue expansion, thereby improving effective spatial resolution without specialized super-resolution optics [27]. This principle has since been adapted to multiple imaging-based spatial transcriptomic workflows. For example, expansion-assisted MERFISH increases the measurable density of high-abundance RNA libraries by spatially separating otherwise overlapping RNA signals [25], whereas ExSeq combines physical expansion with in situ sequencing to map RNAs with nanoscale precision in intact tissues [12]. Expansion-assisted iterative fluorescence in situ hybridization (EASI-FISH) further extends this logic to thick tissue volumes, and recent anchoring chemistries have improved the compatibility of expansion-based imaging with both RNA and protein detection [28,29]. Thus, expansion-based strategies should be viewed not as a MERFISH-specific solution but as a general physical strategy for reducing optical crowding, although they also introduce additional requirements for tissue processing, isotropic expansion, and cross-cycle registration.

Another route is to redistribute transcript signals across a larger coding space so that fewer molecules are observed per image. seqFISH+ implements this through pseudocolor-based dilution, in which transcripts are distributed across a much larger set of sequentially imaged pseudocolors within each fluorescence channel, thereby alleviating density constraints that had limited earlier high-plex implementations [9].

A more conservative alternative is to limit multiplexity, favoring robustness and tissue-scale mapping over maximal throughput. osmFISH adopts a non-barcoded cyclic smFISH design in which each cycle directly measures a limited set of transcripts, simplifying analysis and avoiding some of the overlap-related decoding ambiguities associated with combinatorial barcode readout in dense samples [10].

### 2.3. In Situ Sequencing and Amplified Readout Strategies in Imaging-Based Spatial Transcriptomics

While the above smFISH-derived platforms improved mainly through combinatorial coding and density management, another important technique goes beyond traditional imaging-based methods and incorporates in situ sequencing for signal amplification [11,12]. These approaches have shifted molecular identification from sparse barcode readouts of unamplified RNA puncta to amplified, sequencing-oriented readouts within intact tissues [11,12].

Notably, in imaging-based spatial transcriptomics, the terminology around ISS has moved beyond its original meaning. In a narrow sense, ISS refers to enzymatic base-by-base or ligation-based sequence readout within intact tissue [6,11]. In the context of spatial transcriptomics, however, the term is often used to describe the key ISS protocols that are employed for amplification purposes, including padlock/RCA-assisted methods, while the transcript identity is recovered by cyclic hybridization or barcode decoding rather than by conventional base-by-base sequencing [13,14,15,16]. That is, ISS-based approaches combine ISS amplification with imaging readout in this context. In this review, we therefore use amplification- or ISS-related imaging to refer to this technique while intentionally specifying sequencing-based signal readouts where necessary.

STARmap exemplifies this direction by integrating hydrogel-tissue chemistry, targeted amplification, and in situ sequencing by ligation to profile RNA molecules in three-dimensional tissue contexts [11]. Unlike unamplified smFISH-based methods, STARmap detects amplified transcript-derived products, generating high-intensity signals that are compatible with volumetric imaging in thick samples [11]. More recently, Deep-STARmap has extended this strategy through scalable probe synthesis, hydrogel-based probe anchoring, and cDNA amplicon stabilization, enabling 3D spatial transcriptomic profiling in 60–200 μm thick tissue blocks [30]. A complementary extension, ExSeq, combines in situ sequencing with expansion microscopy, thereby improving effective spatial resolution and molecular accessibility for enzymatic reactions within intact specimens [12]. This enables nanoscale and subcellular readout in complex tissues and supports both targeted and untargeted analyses, including the detection of retained introns, splice variants, and transcript localization within dendrites and spines [12].

Importantly, this amplified-readout family has expanded beyond the original sequencing-by-ligation framework. HybISS uses padlock probes, rolling circle amplification, and sequencing-by-hybridization chemistry to improve robustness and scalability for larger targeted panels in mouse and human brain tissues [13]. Direct RNA-targeted HybISS further bypasses reverse transcription by probing RNA molecules directly before RCA-based amplification and hybridization readout [31]. Commercial platforms such as Xenium similarly fall within this broader ISS-based imaging category and have motivated the systematic benchmarking of platform performance, segmentation, and downstream analysis workflows [14]. More recent methods further diversify this space: RAEFISH combines reverse-padlock amplification with multiplexed fluorescence in situ hybridization (FISH)-based decoding to achieve sequencing-free, genome-scale spatial transcriptome imaging, whereas PRISM uses padlock/RCA products and color-intensity barcodes to enable high-plex RNA imaging in a single imaging round on conventional microscopes [15,16].

Computationally, amplification- and ISS-related platforms are better viewed as assay-specific variants within a broader family of cyclic image-decoding problems, rather than as a fully separate computational regime. Their primary image features are often still spot-like objects: unamplified RNA puncta in smFISH-derived assays, or brighter and often larger RCA amplicons or rolonies (rolling circle colonies) in ISS-related assays. Therefore, many workflows begin with spot or amplicon detection, followed by molecular identity assignment from sequencing cycles, sequence-by-hybridization traces, or barcode patterns of amplified products [11,12]. The distinction is therefore not simply sparse-codebook matching versus non-sparse coding, or spot-based versus pixel-based decoding. Instead, decoding difficulty depends on signal morphology, amplicon brightness and density, cycle structure, registration accuracy, background, and assay-specific error models. Accordingly, decoding strategies can overlap across assay families, including spot-based probabilistic approaches such as PoSTcode and joint localization/decoding or deconvolution-aware approaches such as in situ transcriptomics decoding by deconvolution (ISTDECO) [32,33].

### 2.4. Extending Imaging-Based Spatial Transcriptomics to Complex Specimens and Multimodal Readouts

Early imaging-based spatial transcriptomic platforms were developed primarily using thin or otherwise technically tractable samples, which limited their direct applicability to thick tissues, pathology specimens, and multimodal measurements [10,14]. A later phase of the field, therefore, shifted from increasing multiplexity alone toward expanding assay compatibility with more complex specimens, broader molecular readouts, and more standardized analytical workflows [19,34,35].

One major line of development was the adaptation of transcript imaging for volumetric specimens. The thick-tissue three-dimensional MERFISH combines confocal optical sectioning, deep-learning-assisted image enhancement, and protocols optimized for volumetric specimens to profile transcripts in brain sections up to approximately 200 μm thick [19].

A parallel line of development was the integration of transcriptomic readouts with protein and pathology information in the same specimen. STARmap PLUS exemplified this shift by combining high-resolution spatial transcriptomics with protein localization in intact tissue, thereby linking cellular transcriptional states with histopathological features in an Alzheimer’s disease model [34]. Pathology-compatible platforms have further extended this trajectory to clinically relevant materials. Spatial molecular imaging, including the CosMx/SMI platform, enables automated high-plex RNA and protein imaging at subcellular resolution in formalin-fixed paraffin-embedded (FFPE) tissue samples and supports morphology-informed cell segmentation [35]. In parallel, Xenium represents an ISS-based pathology-compatible commercial imaging platform for subcellular-resolution mapping of targeted gene panels and has prompted the systematic benchmarking of platform performance, segmentation, quality control, and downstream analysis workflows [14].

However, compatibility with FFPE or clinical specimens does not by itself guarantee clinical robustness. Pre-analytical variables, including fixation time, tissue processing, storage duration, deparaffinization, antigen retrieval, RNA fragmentation, and autofluorescence, can introduce substantial variability in signal intensity, probe accessibility, background levels, and apparent detection efficiency [35,36,37]. These factors are especially important for clinical pathology implementation, where samples are collected and processed across different institutions, instruments, and archival conditions. Pathology-aware imaging-based spatial transcriptomics, therefore, requires not only compatible chemistry but also standardized sample handling, explicit quality control (QC) metrics, and batch-aware computational analysis [14,35].

Multimodal pathology workflows further introduce co-registration challenges. When RNA signals, protein markers, hematoxylin and eosin (H&E) staining, and functional imaging are acquired from the same or adjacent tissue sections, the relevant problem is not only round-to-round alignment but also cross-modal registration between signals with different contrast mechanisms, spatial resolutions, staining-induced distortions, and tissue deformation patterns [34,35,38]. Algorithmically, this requires fiducial- or morphology-guided alignment, deformable registration when local distortions are present, and validation that transcriptomic features remain spatially consistent with histological structures after registration [34,35,38].

Other emerging systems have broadened the field in complementary ways. Light-Seq linked imaging with ex situ sequencing through optical selection and spatial indexing of fixed cells or regions of interest, whereas multi omic single-scan assay with integrated combinatorial analysis (MOSAICA) explored alternative encoding and readout strategies for multiplexed RNA and protein detection in clinically relevant specimens [39,40]. Collectively, these developments have shifted imaging-based spatial transcriptomics from a primary focus on transcript multiplexity toward specimen compatibility, multimodal integration, pathology awareness, commercial scalability, and translational use [19,34,35,39,40].

## 3. Data Processing from Images to Molecule

**Figure 3:**
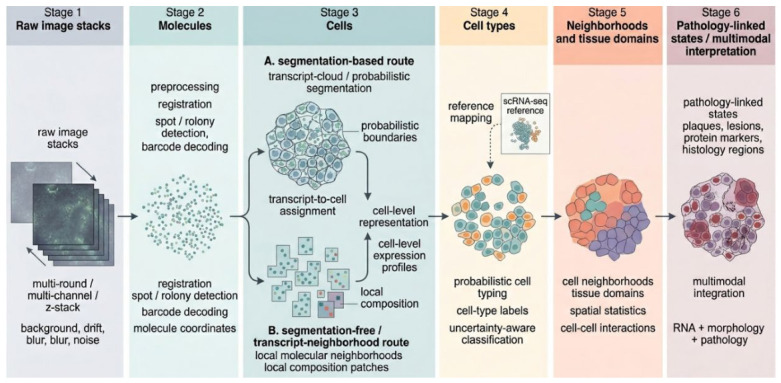
Overview of the common analytical workflow for imaging-based spatial transcriptomics. Raw multi-round image stacks are transformed across four scales: from molecular coordinates (preprocessing and decoding) to cell-level representations (segmentation) and cell-type identities (classification), finally reaching tissue-level and multimodal interpretations.

The first computational stage in imaging-based spatial transcriptomics converts multi-round fluorescence or in situ sequencing images into a molecular representation through preprocessing, registration, restoration, feature detection, and decoding. Despite differences in assay chemistry and optical design, the central objective is the same: to recover a reliable molecular point cloud that can support downstream cell- and tissue-level inferences [5,11,12] (Figure 3).

### 3.1. Image Preprocessing and Quality Control

The input for imaging-based spatial transcriptomics is typically a collection of multi-round, multi-channel, and often volumetric fluorescence images, rather than a cell-by-gene matrix. Therefore, image preprocessing is the necessary starting point for downstream molecular inference. Across different platforms, raw images are affected by uneven illumination, diffuse or autofluorescent backgrounds, photobleaching, focus variability, and specimen-dependent optical artifacts, all of which can distort subsequent molecule detection and decoding [9,10,19].

Beyond these image-level artifacts, the scale of the raw data itself presents a computational bottleneck. Multi-round, multi-channel, and three-dimensional acquisitions can generate hundreds of gigabytes to terabytes of images per specimen, making storage layout, compression, metadata tracking, and input/output efficiency important components of the analytical workflow rather than merely engineering details [41,42]. Modern bioimage formats and spatial omics data frameworks increasingly address this problem through chunked, multiscale, cloud-compatible storage and lazy access to larger-than-memory arrays, as exemplified by OME-Zarr/OME-NGFF and SpatialData [41,42,43]. For imaging-based spatial transcriptomics, however, compression and downsampling must be used cautiously because weak punctate signals from low-abundance transcripts can be biologically meaningful and may be lost if storage optimization is decoupled from molecule-detection performance [9,19].

After these data-management and QC considerations, preprocessing operations still need to be tailored to the assay design and specimen type. In cyclic planar imaging workflows, preprocessing often focuses on correcting illumination nonuniformity and structured backgrounds. For example, osmFISH incorporated illumination correction, background estimation, and background subtraction before spot detection [10]. In dense or volumetric datasets, preprocessing becomes even more consequential because out-of-focus fluorescence, spherical aberration, and depth-dependent signal attenuation can alter the apparent abundance and localization of transcripts [9,19]. In thick-tissue three-dimensional MERFISH, confocal imaging improves optical sectioning but reduces photon collection at short exposure times; learning-based restoration is used to improve the signal-to-noise ratio and partially recover the detection performance achieved with longer exposures [19].

Quality control should also be coupled to the imaging process itself rather than being treated as a downstream formality. At a minimum, preprocessing quality should be assessed through fiducial stability, signal-intensity distributions across rounds and channels, background characteristics, and the reproducibility of detected molecular features across fields of view or replicate samples [9,10,19]. Such QC criteria are increasingly supported by dedicated computational frameworks. For example, MerQuaCo quantifies common imperfections in image-based spatial transcriptomics data, including tissue damage, tissue detachment, dropped images, transcript-density variation, and depth-dependent detection heterogeneity [44], whereas SpatialQC provides a broader spatial transcriptomics QC workflow for cell/spot scoring, data filtering, and interactive report generation [45]. Related open-source resources, such as SpaceTrooper and SegTraQ, further illustrate the movement toward practical, reproducible QC and segmentation-assessment workflows. Because the analytical pipeline is cumulative, errors introduced at this stage are not confined to image quality alone: overcorrection can suppress low-intensity molecules, residual background can inflate false detections, and depth-dependent artifacts can later be misinterpreted as biological variation [9,19].

### 3.2. Registration and Alignment

Cyclic imaging methods repeatedly image the same physical field across multiple rounds, making registration integral to molecular identity recovery, rather than a simple image alignment step. In imaging-based spatial transcriptomics, molecular identity is often inferred by linking signals across rounds or channels; consequently, even small spatial misalignments can corrupt barcode decoding or molecule assignment [5,9,11]. The central task is therefore to correct round-to-round drift and channel-specific chromatic offsets so that each molecular feature is mapped to a consistent spatial coordinate system throughout the assay [5,9,11].

The exact mathematical formalization of this problem depends on the platform design. In MERFISH and seqFISH-like workflows, registration is commonly performed using fiducial-based translational, rigid, or affine alignment across imaging rounds and fluorescence channels, together with chromatic correction, so that the same RNA molecule can be localized consistently over repeated hybridization cycles [9,18,19]. In amplification-assisted in situ sequencing methods such as STARmap, the corresponding task is to align sequencing cycles so that the same transcript-derived amplicon can be read out as a coherent identity over repeated rounds; when sample distortion is non-negligible, affine registration may need to be supplemented by local deformable correction [11,18]. In volumetric imaging, registration may also need to account for Z-axis drift and depth-dependent optical distortion, which become more prominent in thick tissues [19]. Registration errors are particularly important in cyclic imaging because misalignment between rounds or channels can, in principle, assign signals from different molecular features to the same barcode trace [5,9,11,19].

Therefore, many platforms rely on fiducial-based stabilization and integrated imaging workflows to support large-scale cyclic acquisition. CosMx/SMI, for example, uses fixed fiducials as an optical reference across readout cycles [35]. In practice, registration quality should be assessed not only by visual image overlap but also by assay-level diagnostics, including fiducial residuals, the cross-round matching of stable features, apparent molecule recovery, barcode consistency, blank-code behavior when available, and reproducibility across fields of view or replicate samples [5,9,11,19,35].

### 3.3. Deconvolution and Denoising for Image Restoration

As imaging-based spatial transcriptomic datasets have become denser and have extended to thicker specimens, image restoration has become increasingly important for accurate molecule recovery [9,19]. In this context, image restoration broadly includes operations such as deconvolution and denoising that improve feature separability before detection and decoding [9]. Deconvolution seeks to reverse optical blur, typically by modeling the imaging point-spread function, whereas denoising suppresses stochastic noise and background fluctuations [46]. In dense transcript fields, these operations can improve feature separability by sharpening punctate signals, partially resolving overlapping signals, and increasing effective contrast for localization and decoding [9]. In thick-tissue three-dimensional MERFISH, restoration plays an even more explicit role: fast, low-exposure confocal images showed reduced transcript detection performance, and learning-based enhancement was used to improve the signal-to-noise ratio and partially recover the detection performance achieved with longer exposures [19]. These developments indicate that assay throughput and molecular sensitivity are increasingly shaped not only by optics and chemistry but also by the computational restoration pipeline [19].

In practice, restoration can be implemented through several complementary approaches. Classical model-based approaches include Richardson–Lucy and Wiener deconvolution, whereas denoising methods such as non-local means, wavelet-based filtering, and BM3D are often used to suppress stochastic noise, structured background, or photon-limited fluctuations before feature detection [46,47]. These denoising methods are attractive because they are training-free, relatively transparent, and can be applied without paired low- and high-signal-to-noise ratio (SNR) images. However, their performance depends strongly on noise assumptions and parameter settings; excessive smoothing or inappropriate filtering can attenuate weak RNA puncta, alter spot intensity distributions, or reduce the separability of nearby molecules. Recent open-source implementations have made deconvolution more practical for large fluorescence datasets: RedLionfish provides GPU-accelerated Richardson–Lucy deconvolution for volumetric data, whereas Deconwolf enables high-performance deconvolution of crowded FISH and imaging-based spatial omics images [48,49]. Deconwolf also illustrates that restoration can affect downstream molecular readouts, increasing transcript recovery in in situ spatial transcriptomics and improving downstream cell-type assignment after deconvolution [49]. In parallel, learning-based image restoration methods have expanded the restoration toolbox. For example, content-aware image restoration (CARE) uses a U-Net-like neural network to learn mappings from low-SNR to high-SNR fluorescence images and can support denoising, deconvolution-like restoration, or resolution enhancement when representative paired or simulated training data are available [50]. The joint sparse method for imaging transcriptomics (JSIT) further illustrates how restoration and decoding can be coupled within a single optimization framework. JSIT models imaging-based spatial transcriptomics data using a point-spread-function-aware image formation model and uses sparsity constraints together with codebook information to jointly infer transcript locations and molecular identities [51]. Conceptually related methods further illustrate this restoration-aware decoding logic: BarDensr uses a physical observation model and sparse non-negative regression to estimate demixed rolony densities, whereas ISTDECO combines spatial and spectral deconvolution to jointly infer barcode identity and location [33,52]. The general concept of barcode decoding is discussed further in Section 3.5.

Simultaneously, restoration methods introduce interpretive risks. Overaggressive denoising or deconvolution can alter spot morphology, distort intensity distributions, or generate puncta-like artifacts that bias downstream detection [9,19]. These risks are particularly important for learning-based restoration because neural networks can generalize poorly across imaging conditions or hallucinate plausible but unsupported image details when training data are insufficient, biased, or mismatched to the target experiment [53]. Restoration in spatial transcriptomics should therefore be evaluated not only by visual image quality but also by downstream molecular criteria, such as molecule recovery, decoding consistency, blank-code behavior when available, downstream cell-type assignment, and reproducibility across fields of view or replicate samples [9,19,49]. In practice, restoration is most useful when treated as part of the measurement model rather than as a generic image-enhancement step [19].

### 3.4. Detection of Punctate or Amplified Molecular Features

After preprocessing and registration, molecular feature detection is the key step that converts the image signal into candidate molecular objects. In hybridization-based assays, these objects are typically puncta or spots corresponding to individual RNA molecules or barcode readout events [9,10]. In amplification-assisted in situ sequencing workflows, by contrast, the relevant objects are locally amplified products, often referred to as amplicons or rolonies, which are generally brighter and more spatially extended than unamplified single-molecule puncta [11,12]. This stage, therefore, converts fluorescence images into a candidate molecular point cloud for downstream decoding, quantification, and spatial analysis [9,10,11,12].

The difficulty of detection depends strongly on the assay design, object morphology, and signal density. In osmFISH, spot detection is performed using Laplacian-of-Gaussian enhancement followed by local-maximum detection and data-derived thresholding, illustrating a classical model-based strategy for identifying punctate signals in cyclic imaging data [10]. In seqFISH+, dense transcript packing remains a central challenge even after experimental density redistribution, so localization-based processing is required to separate closely spaced signals [9]. In amplification-assisted platforms such as STARmap, the measured objects are transcript-derived amplicons rather than single-molecule spots, and detection must therefore account for both signal intensity and feature morphology [38]. Importantly, morphology-aware detection is not limited to amplicon-based assays: radial-symmetry-based methods such as RS-FISH exploit the approximately round geometry of diffraction-limited FISH spots and have been used in large 2D/3D FISH image volumes, including cleared or expanded samples [54]. Thick-tissue workflows such as EASI-FISH further illustrate the need for scalable spot detection in large volumetric datasets [28]. In parallel, deep learning-based methods are increasingly applied to large, low-SNR datasets: Spotiflow formulates spot detection as subpixel-accurate deep stereographic flow regression, whereas Polaris integrates weakly supervised spot detection into an image-based spatial transcriptomics analysis pipeline [55,56]. Other neural-network-based tools, such as deepBlink and U-FISH, further show that learned spot detection depends on representative annotated or pseudo-annotated training data and should be validated across SNR regimes, imaging modalities, and tissue contexts [57,58]. These detection strategies involve different trade-offs. Laplacian-of-Gaussian filtering and local-maximum detection, as used in osmFISH, provide a simple, interpretable, and efficient strategy for sparse, high-SNR puncta, but their performance can be sensitive to threshold selection, uneven background, and optical crowding [10]. Radial-symmetry-based methods such as RS-FISH can achieve accurate subpixel localization by exploiting approximately round point-spread-function geometry, although this assumption may become less appropriate for strongly overlapping signals, anisotropic optical blur, or morphologically irregular amplified features [54]. Deep learning-based detectors can improve robustness in low-SNR or heterogeneous images, but their performance depends on representative training data, annotation quality, and cross-platform generalization [55,56,57,58]. Community benchmarking efforts in single-molecule localization microscopy further show that method ranking depends on evaluation criteria such as localization error, recall, precision, the Jaccard index, and false-positive or false-negative behavior; therefore, detection algorithms in imaging-based spatial transcriptomics should be selected and validated according to the assay design and downstream molecular-calling task rather than by visual image quality alone [59].

Detection quality directly shapes the molecular representation of the assay. Missed features generate false negatives, spurious detections produce false positives, and merged or split objects distort both abundance estimates and downstream identity recovery [9,10,11,12,55,56]. These errors are especially consequential because they are often propagated into later stages as apparent biological structures. Detection should therefore be evaluated not only by visual image quality, but also by whether the resulting feature set behaves as a plausible molecular point cloud under the assay design, coding scheme, and available controls [9,10,11,12].

### 3.5. Barcode Decoding and Molecule Calling

Barcode decoding converts multi-round observations into molecular identity. Although the exact implementation varies across different platforms, the task generally takes one of three forms: codebook matching of binary readout patterns in MERFISH, localization matching and pseudocolor assignment in seqFISH-like systems, or sequencing-style decoding of multicycle color patterns in amplification-assisted methods such as STARmap and ExSeq [5,9,11,12]. In each case, molecular identity is recovered from repeated observations distributed across imaging or sequencing cycles, rather than from a single image alone.

Despite these platform-specific differences, the underlying problem is similar: an observed multi-round signal trace must be mapped to a valid molecular identity under incomplete and noisy measurements [5,9,11,12]. Therefore, decoding performance depends jointly on codebook design, alignment accuracy, feature detection, and the statistical structure of assay-specific noise [5,9,11,12]. In multi-round hybridization assays, cycle-dependent signal loss caused by photobleaching, incomplete hybridization, or inefficient stripping can convert expected “on” bits into missing observations or introduce residual signals across cycles, making barcode decoding sensitive to both the optical stability of the fluorophores and the error tolerance of the codebook [5,9,60]. Molecule calling is the step in which candidate multi-round signals are evaluated and either accepted as molecules or discarded according to assay-specific quality criteria. In practice, this usually involves dictionary matching or sequence assignment, rejection of invalid or low-confidence patterns, and additional filtering to suppress spurious identifications [5,9,11,12]. When available, blank or null barcodes provide a direct empirical control for estimating misidentification rates, whereas other pipelines rely on quality thresholds, cycle-level consistency rules, or assay-specific filters [5,60].

This stage makes it particularly clear that assay performance is co-determined by chemistry and computation. Better codebooks are important for unambiguous decoding, while improved alignment reduces false correspondences, and more effective physical separation lowers ambiguity before decoding begins [5,9,11,12,60]. Some integrated analysis pipelines clearly showcase such dependencies; for example, Polaris couples learned spot detection with probabilistic gene decoding rather than treating the two as fully independent stages [56]. Barcode decoding and molecule calling thus define the transition from an imaging signal to a spatial molecular representation and, in doing so, shape all downstream analyses [5,9,11,12,56,60].

## 4. Data Interpretation from Molecules to Cells and Tissue Organization

### 4.1. Cell Segmentation and Transcript-to-Cell Assignment

Once a molecular point cloud is recovered, the spatial expression matrix becomes available at its finest single-molecule resolution. However, this resolution is often not informative because it is highly sparse and does not assign molecules to specific cells. Therefore, the first challenge for data interpretation is to convert the molecular coordinates into cell-resolved measurements, i.e., cell segmentation and transcript-to-cell assignment. Through this procedure, a cell-by-gene matrix can be derived, which is crucial for many downstream analyses, including, but not limited to, cell typing, differential expression, spatial-domain inference, and cell–cell interaction analysis [10,17,18]. Early studies often relied on nuclei stains, poly(A) stains, or watershed-based segmentation, but these approaches can perform poorly in dense tissues, in cells with elaborate morphologies, or when cellular boundaries are weakly marked [10,18]. In osmFISH, for example, segmentation difficulties in densely packed regions are explicitly noted, underscoring that cell boundary inference is often a limiting factor [10].

A major methodological shift occurred when segmentation began to incorporate transcript coordinates rather than relying solely on cell morphology. The pciSeq algorithm uses a probabilistic framework to extend nuclei-based boundaries while jointly assigning transcripts and cell identities, demonstrating that transcript-to-cell assignment may require uncertainty-aware inference rather than fixed boundaries alone [18]. Baysor made this transition more explicit by modeling cells as spatially coherent molecular distributions with characteristic transcriptional compositions, optionally integrating auxiliary staining data, but not requiring it [17]. More recent methods have extended this direction in a different manner. Proseg uses unsupervised probabilistic modeling of transcript spatial distributions to infer morphologically plausible cell boundaries without multimodal staining, whereas CelloType adopts a multitask learning framework that jointly performs segmentation and classification in image-based spatial omics data [61,62]. Bering further advances this direction by using transferred graph embeddings to jointly perform noise-aware cell segmentation and annotation while exploiting transcript colocalization structure in 2D and 3D spatial transcriptomics data [63]. Because these segmentation and assignment models rely on different priors, training signals, or reference information, their performance should be evaluated against orthogonal boundary evidence, manually curated annotations, simulated perturbations, or held-out tissues when available. Importantly, validation should not rely only on visual boundary plausibility but also on the stability of the resulting cell-by-gene matrix and downstream cell-type or spatial-domain assignments [17,61,62,63]. Table 1 summarizes representative computational tools for cell segmentation and transcript-to-cell assignment in imaging-based spatial transcriptomics.

Transcript-to-cell assignment is a companion problem to segmentation and should not be treated as a secondary implementation detail. Even when a putative cell outline is available, peripheral processes, overlapping structures, sparse membrane markers, or partial sectioning can make transcript assignment ambiguous [17,18,61,62,63]. This is especially problematic in neurons, immune infiltrates, and thick tissues, where cell morphology is extended or irregular; even transcript-aware or learned segmentation methods may fail to accurately recover thin neurites, branched processes, activated immune cell protrusions, or overlapping boundaries in dense tissue [17,61,62,63]. Because segmentation and assignment errors propagate directly into the cell-by-gene matrix, they can generate artifactual cell states, blur true biological differences, and distort downstream analyses as substantially as genuine biological variation [17,18,61,62,63]. Such matrix-level distortions can further affect differential expression testing, spatial-domain inference, neighborhood composition analysis, and cell–cell interaction estimates because all of these analyses treat the cell-by-gene matrix as their primary input [17,18,61,62,63]. Recent methods, therefore, attempt to mitigate this error propagation either by refining segmentation and transcript assignment or by correcting the resulting expression matrix. For example, FastReseg uses transcript-level likelihood scores to identify spatial doublets and misassigned transcript groups, enabling local reassignment, removal, or refinement while preserving the initial image-based segmentation [64]. By contrast, resolVI operates downstream of segmentation by modeling observed cell-level expression as a mixture of true expression, neighboring-cell diffusion, and background, thereby reducing spurious co-expression after quantification [65]. DenoIST similarly addresses post-segmentation transcript contamination in image-based spatial transcriptomics, using a neighborhood-aware Poisson mixture model to identify and remove contaminating transcripts while preserving integer count interpretation [66]. Xenium-focused benchmarking and signal-purification approaches, such as spatial purification of layered intracellular transcripts (SPLIT), further highlight that the segmentation strategy, transcript spillover, and cell-level QC should be evaluated jointly rather than treating transcript assignment as a fixed output of a platform pipeline [67]. More broadly, spatial denoising frameworks such as SpaceBender illustrate that local ambient or diffused RNA signals can be modeled to enhance biological signals in both spot-resolution and subcellular-resolution spatial transcriptomic data, including MERFISH, CosMx, and Xenium datasets [68]. Together, these methods emphasize that cell-level expression matrices should be viewed as uncertainty-bearing outputs of segmentation and transcript assignment rather than as error-free measurements.

### 4.2. Segmentation-Free Analysis and Local Molecular Composition

Not all analyses require the finalization of cell boundaries. In high-resolution spatial transcriptomic data, local transcript neighborhoods can encode substantial biological structures even before explicit segmentation is achieved [17,69]. This observation has motivated segmentation-free strategies that operate directly on molecular coordinates or local transcript compositions rather than on predefined cells [17,69].

A representative example is the neighborhood composition vector framework introduced in the Baysor study, in which local molecular neighborhoods are summarized by the relative frequencies of nearby transcripts and used as spatially indexed pseudo-cell representations [17]. Such representations can reveal tissue organization, local mixtures of cell states, and patch-like molecular structures (including membraneless organelles) even when cell boundaries remain uncertain [17]. A similar idea has subsequently been extended to broader classes of high-resolution spatial transcriptomic data. For example, factor inference of cartographic transcriptome at ultra-high resolution (FICTURE) applies scalable spatial factorization to infer local molecular patterns without requiring explicit cell segmentation [69]. These approaches are particularly useful in dense tissues, fragmented specimens, or subcellular-resolution datasets in which transcript density is sufficient to reveal local organization but not necessarily sufficient to support robust cell boundary inference [17,69].

Segmentation-free analysis is not a substitute for all cell-resolved tasks, but it provides an important complementary representation when segmentation is uncertain, incomplete, or biologically ill-posed. It is especially useful for identifying spatial domains and molecular niches, where the analytical target is the local structure rather than a finalized cell-by-gene matrix alone [17,69]. For this reason, modern imaging-based spatial transcriptomics should not be framed as a choice between “correct segmentation” and “failed segmentation” but as a spectrum of molecular representations with different analytical resolutions and assumptions [17,69].

### 4.3. Cell Typing, Reference Mapping, and Probabilistic Classification

Cell typing in imaging-based spatial transcriptomics has evolved from marker-based heuristics to reference-guided and probabilistic inference. Early targeted-panel studies often clustered measured cells de novo and assigned identities using canonical marker genes, an approach that remains useful when spatial assays reveal populations that are spatially distinctive or incompletely represented in sequencing references [10,18]. However, as single-cell RNA-sequencing (scRNA-seq) atlases have matured, spatial cell typing has increasingly shifted toward reference integration or reference mapping, in which targeted in situ measurements of one cell in the spatial transcriptomics data are integrated with (or mapped onto) the most similar cells in the reference single-cell atlas of the same tissue type to infer cell types [10,17,18].

**Figure 4:**
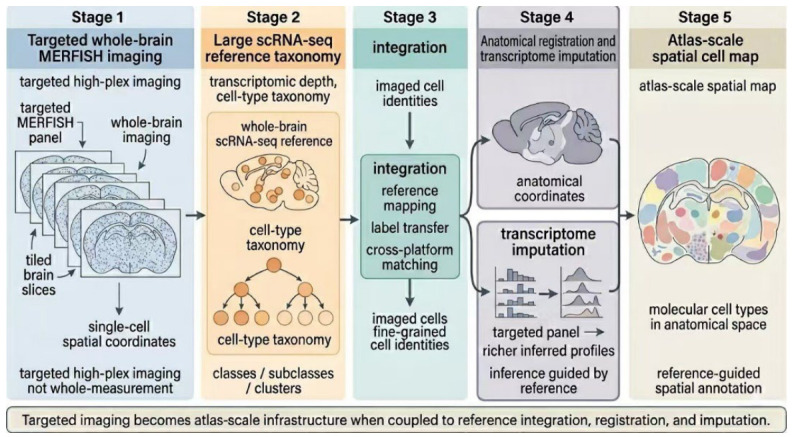
Reference-guided integration for atlas-scale spatial annotation. Targeted MERFISH provides spatial context for specific gene panels, while scRNA-seq references offer more comprehensive gene expression characteristics of a specific cell type. Through a pipeline of label transfer, cross-platform matching, and transcriptome imputation, targeted imaging data is transformed into atlas-scale maps that anchor molecular cell types within their anatomical space.

A canonical example is the pciSeq algorithm, which was designed to assign cells probabilistically to scRNA-seq-defined types from limited in situ panels and is particularly useful when the central task is to distinguish closely related subtypes rather than broad classes alone [18]. Later atlas-scale studies combined targeted spatial measurements with large single-cell references and used label transfer, nearest-neighbor mapping, or related integrative strategies to assign fine-grained identities to cells. In the whole-brain MERFISH atlas, MERFISH measurements are integrated with scRNA-seq using canonical correlation analysis followed by k-nearest-neighbor classification, enabling subclass- and cluster-level annotation together with transcriptome-wide imputation [20]. Similarly, the CNS STARmap PLUS atlas uses Harmony-based integration and nearest-neighbor label transfer to derive molecular cell-type maps across the adult mouse central nervous system [70,71] (Figure 4). More broadly, reference-mapping approaches, including Harmony-based integration, anchor-based transfer, and nearest-neighbor classification, are now widely used to connect spatial profiles with single-cell atlases across tissues and disease contexts [72]. In heterogeneous tumor systems, for example, single-cell and spatial transcriptomic data have been jointly used to reconstruct cell states, spatial niches, and tumor–microenvironment interactions [73]. However, reference-dependent annotation can bias interpretation toward predefined cell states, propagate reference errors, and reduce sensitivity to rare, transitional, or context-specific transcriptional programs. Therefore, transferred labels should ideally be evaluated together with marker expression, spatial localization, confidence scores, and unsupervised or de novo analyses [74].

This shift is conceptually important because it changes the interpretation of targeted spatial panels. A panel is no longer merely a restricted measurement; instead, it can serve as an anchor into a richer transcriptomic reference framework, provided that the selected genes preserve discriminative structure and that the integration strategy is appropriately validated [18,20,70]. At the same time, this strategy makes spatial interpretation partly dependent on the reference used for annotation, because transferred labels inevitably reflect the assumptions, resolution limits, and biases of that reference taxonomy [18,20,70]. For this reason, cell typing in imaging-based spatial transcriptomics is often best understood as a confidence-weighted inference rather than direct label recovery. Beyond count-based reference mapping, recent work has also begun to incorporate spatial context and subcellular transcript organization into classification itself; a recent multiscale topological framework is one example of this direction [75].

### 4.4. Spatial Domains, Tissue Regions, and Atlas Alignment

As imaging-based spatial transcriptomics has matured, analysis has increasingly moved beyond spatially resolved cell typing toward the inference of higher-order tissue structures. Spatial domains, anatomical regions, and continuous gradients cannot always be reduced to cell-type labels alone, because tissues often exhibit region-specific organization, boundary transitions, and local compositional patterns that emerge only at the neighborhood level [9,10,20,70].

In an osmFISH-based study, clustering was combined with spatial statistics to reconstruct anatomical organization and quantify preferential colocalization or avoidance among cell types in the somatosensory cortex [10]. In another study using the seqFISH+ platform, hidden Markov random field-based analysis was used to identify spatial domains that are not fully explained by cell-type assignments alone [9]. Atlas-scale studies have extended this logic further, but their value is not limited to cell-type mapping. In the whole-brain MERFISH atlas, registration to the Allen Common Coordinate Framework has enabled the identification of spatial modules and gradients, highlighting that tissue organization is often continuous rather than strictly partitioned into discrete regions [20]. Similarly, the CNS STARmap PLUS atlas infers molecular tissue regions from spatial niche gene expression rather than relying solely on classical anatomical boundaries [70].

A related but distinct challenge is to place inferred spatial domains and tissue regions into shared anatomical and transcriptomic reference frameworks. Spatial transcriptomics at the atlas scale depends not only on molecular detection but also on alignment to common coordinate systems and reference taxonomies. In large brain atlases, these steps are essential for making spatial maps comparable across animals and interpretable within established anatomical and cell-type hierarchies [20,70]. More recent computational frameworks, such as STIM, further show that visualization, alignment, and three-dimensional reconstruction are not merely presentation steps but analytical components for integrating serial spatial transcriptomic sections into spatially consistent three-dimensional reconstructions [38].

### 4.5. Spatial Interaction Analysis and Higher-Order Tissue Interpretation

Once cells or local molecular neighborhoods have been placed into spatial context, the inference of interaction becomes essential for interpreting tissue and spatial niche organization, including how neighboring cells, local environments, and recurrent spatial motifs shape tissue function or pathology [20,35,70].

Pathology-oriented platforms have been particularly useful in illustrating this transition. Spatial molecular imaging/CosMx incorporated neighborhood and niche analysis into high-plex RNA–protein workflows in FFPE tissue sections, enabling the characterization of associations among cell states, tumor–microenvironment structures, and putative ligand–receptor interaction patterns [35]. STARmap PLUS similarly linked transcriptomic states with histopathological features in Alzheimer’s disease, supporting the interpretation of plaque-centered shells and disease-associated cellular microenvironments as biologically meaningful spatial configurations [70]. These examples show that the relevant spatial objects are not only individual cells but also recurrent multicellular neighborhoods, lesion-centered niches, and pathology-associated spatial states [35,70].

Beyond such pathology-oriented examples, dedicated computational frameworks have begun to formalize interaction and niche inference more explicitly. Methodologically, the field is moving from simple pairwise proximity and simple ligand–receptor scoring to modeling of higher-order spatial organization. Recent approaches, such as CellNEST, use attention-based modeling to infer not only pairwise communication but also relay-like interaction structures in spatial transcriptomic data [76]. Foundation-model-based approaches are beginning to extend this direction further. Nicheformer, for example, learns transferable niche-aware embeddings from single-cell and targeted spatial transcriptomic data, illustrating how local microenvironment modeling may increasingly become a representation-learning problem rather than a purely post hoc analytical step [77]. These advancements facilitate better interpretation of the spatial organization of a tissue, especially in the context of an imaging-based spatial transcriptomics platform, where typical receptor–ligand pairs are not always included in the gene panel.

## 5. Biomedical Applications

### 5.1. Subcellular RNA Organization

A distinguished feature of imaging-based spatial transcriptomics is that it resolves not only which transcripts are expressed but also where they are localized within cells [4,9,12,60]. This capability is especially important when the biological question concerns intracellular organization rather than cell identity or tissue composition alone [4,9,12,60].

MERFISH and seqFISH+ make this especially clear at this scale. Near-transcriptome-scale MERFISH combines high-plex RNA profiling with cellular structure imaging to identify transcripts enriched in distinct intracellular compartments, including the nucleus and endoplasmic reticulum, and further uses the nuclear-to-cytoplasmic RNA balance to infer cell-state dynamics in situ [60]. seqFISH+ likewise resolves thousands of genes with distinct subcellular localization patterns, including perinuclear, cytoplasmic, and protrusion-enriched transcript groups [9]. ExSeq extends these analyses into finer subcellular architecture by detecting RNAs in dendritic spines, branches, and other nanoscale compartments in intact tissue [12]. Together, these studies show that imaging-based spatial transcriptomics makes subcellular RNA organization itself a measurable transcriptomic phenotype, thereby enabling the study of localized translation, cellular polarity, and compartment-specific RNA regulation [9,12,60].

### 5.2. Tissue Organization and Spatial Gradients Across Local and Atlas Scales

Through the reconstruction of tissue architecture, transcript-defined cell populations are directly mapped onto anatomical spaces. Early studies showed that targeted marker panels could reconstruct cortical organization from the spatial distributions of clustered cells, demonstrating that tissue atlases could be derived directly from measured molecular distributions rather than from histological annotation alone [10,11,18]. In an osmFISH study, clustering and spatial-statistical analysis recovered the anatomical organization of the mouse somatosensory cortex and quantified preferential colocalization or avoidance among cell types [10]. Similarly, STARmap and MERFISH studies have linked transcriptomic cell identity to cortical lamination, local cellular organization, and projection-related spatial patterns in intact neural tissues [11,78].

Beyond discrete anatomical regions, imaging-based spatial transcriptomics can resolve continuous molecular gradients and spatially restricted developmental programs. In the mouse primary motor cortex, MERFISH links transcriptomic identity to laminar organization, spatial gradients, and projection patterns [78]. Whole-brain MERFISH further showed that molecular tissue organization includes spatial modules and continuous gradients rather than just sharply separated anatomical compartments [20]. Developmental studies have extended this logic by using MERFISH to resolve human fetal cortical layer and area specification and to characterize spatially patterned complement-system organization during postnatal mouse brain development [79,80]. These examples indicate that spatial gradients and developmental patterning are central biological outputs of imaging-based spatial transcriptomics, not merely by-products of cell-type mapping.

At larger scales, atlas-level imaging further integrates cell identity, tissue regions, spatial neighborhoods, and anatomical reference frameworks. Whole-brain MERFISH and the CNS STARmap PLUS atlas enabled atlas-scale mapping of cell types, molecular tissue regions, and spatial neighborhoods across the mouse nervous system, revealing that tissue organization often combines discrete anatomical structures with continuous molecular variation [20,67,81]. Importantly, the value of these atlases is not limited to assigning cell types to coordinates; they also provide frameworks for comparing spatial domains across animals, linking local niches to global anatomy, and interpreting molecular tissue organization across scales [20,67,81]. Together, these studies show that tissue-level applications of imaging-based spatial transcriptomics connect molecular cell states with anatomical organization, spatial gradients, developmental programs, local neighborhoods, and atlas-scale reference systems.

### 5.3. Disease Microenvironments and Pathology-Compatible Spatial Profiling

Tumors and other pathological tissues provide a stringent setting for spatial methods because they combine cellular heterogeneity, architecture-dependent interactions, and the need to preserve histological context in clinically relevant specimens [32,33]. Imaging-based spatial transcriptomics is valuable in this context because it can link molecular states directly to tissue organization while remaining increasingly compatible with pathology-oriented workflows [34,35].

Spatial molecular imaging, including the CosMx/SMI platform, exemplifies this direction by enabling high-plex RNA and protein imaging in FFPE tissues with morphology-guided segmentation, thereby supporting the identification of tumor and stromal cell states, spatial niches, and putative cell–cell interaction patterns in clinically relevant samples [35]. Subsequent studies have applied CosMx/SMI to clinically relevant tumor specimens, including brain tumors and small cell lung cancer, to resolve pathology-linked niches, microbe-associated or immune-associated spatial microenvironments, and treatment-relevant spatial interactions. Beyond tumor profiling, pathology-aware spatial transcriptomics can also resolve lesion-centered disease microenvironments [82,83]. STARmap PLUS, for example, mapped transcriptomic states together with amyloid-β plaques and hyperphosphorylated tau in an Alzheimer’s disease model, revealing spatially structured pathological niches around lesions [35].

Some other platforms point to complementary translational formats rather than a single, dominant workflow. Light-Seq enables imaging-guided spatial indexing of selected cells or regions for downstream sequencing, whereas MOSAICA explores multiplexed RNA–protein profiling in autofluorescent and clinically relevant materials [39,40].

## 6. Computational Challenges and Future Directions

### 6.1. Optical Crowding and Uncertainty Propagation in Spatial Interpretation

A central challenge in imaging-based spatial transcriptomics is that scaling transcript coverage raises molecular density, thereby increasing ambiguity in localization, decoding, and molecule calling [5,9,19]. Optical crowding is not only an imaging limitation but also a source of statistical uncertainty that propagates across the analytical pipeline: missed separations reduce molecule recovery, merged detections distort abundance estimates, and incorrect correspondences alter inferred identities, and these errors can subsequently bias transcript assignment, cell typing, and spatial interaction analysis [5,9,19]. More broadly, image-processing decisions can reshape downstream biological interpretation even when the underlying tissue is unchanged: restoration artifacts may generate or suppress puncta-like signals, detection errors can alter the molecular point cloud, and segmentation or assignment errors can distort the cell-by-gene matrix [17,19,49,58,64,65,67]. Strategies such as error-robust coding, pseudocolor dilution, physical expansion, deconvolution, learning-based detection, and transcript-aware segmentation can reduce this burden, but they do not fully eliminate the need for uncertainty-aware modeling and downstream validation [5,9,17,19,76]. A major future challenge is, therefore, to move from hard molecular and cellular calls toward probabilistic representations that retain confidence information across downstream stages of analysis [5,9,17,18,19,76].

### 6.2. Cell Boundary Inference as a Central Challenge in Cellular-Resolution Interpretation

Cellular-resolution interpretation remains fundamentally limited by cell boundary inference, which remains unreliable in dense tissues, highly polarized cells, and partially sectioned or weakly stained specimens [17,18,61]. The field has moved from nuclei-guided heuristics toward transcript-aware probabilistic and learned models, including Baysor, Proseg, CelloType, and Bering; however, robust segmentation and transcript-to-cell assignment remain unresolved in many complex tissues [17,18,61,62,63]. In particular, thin neuronal or immune cell processes remain difficult to delineate because they may contain sparse transcripts, overlap with neighboring cells, and lack clear membrane or cytoplasmic markers [17,61,62,63]. This is not a minor implementation issue: errors at this stage directly alter the cell-by-gene matrix and thereby affect cell typing, spatial-domain inference, interaction analysis, and downstream biological conclusions. More fundamentally, the challenge is not only where a boundary should be drawn but also how molecules should be attributed to specific cells under intrinsically ambiguous spatial conditions [17,18,61,62,63].

### 6.3. Accuracy for Reference Integration

Reference-guided annotation has greatly expanded the interpretability of targeted spatial panels, especially in atlas-scale studies, but it also introduces new dependencies [20,70]. Spatial labels increasingly inherit the assumptions, resolution limits, and sampling biases of the sequencing taxonomies used for transfer or imputation [20,70]. Simultaneously, panel design itself becomes a source of bias: a targeted assay can function as a robust anchor only if the selected genes preserve the discriminative structure of the reference [20,70]. These issues complicate cross-platform integration because different spatial assays do not always produce the same kinds of molecular, cellular, or regional representations. Segmentation-free frameworks such as FICTURE make this especially clear by showing that biologically meaningful structure can sometimes be recovered without explicit cells, thereby underscoring that cross-platform integration is often a problem of reconciling heterogeneous spatial representations across platforms, resolutions, and assay chemistries rather than simply transferring labels between datasets [69].

### 6.4. Computational Challenges in 3D, Multimodal, and Pathology-Aware Spatial Analysis

Three-dimensional, multimodal, and pathology-aware spatial transcriptomics amplify many core computational difficulties in the field, but they do so in different ways. In thick tissues, signal attenuation, aberration, out-of-focus backgrounds, and complex three-dimensional morphology complicate restoration, registration, and transcript assignment [19,35]. These depth-dependent artifacts are not always spatially uniform: signals from deeper tissue layers may show reduced intensity, broadened point-spread functions, lower signal-to-background ratios, and increased cycle-to-cycle variability, which can affect both molecule recovery and barcode decoding [19,30]. Learning-based restoration can partially compensate for low-SNR or depth-degraded images, but it should not be treated as an unbiased replacement for optical measurement; hallucinated puncta, suppressed weak signals, or depth-specific overcorrection can directly distort molecule calls and downstream biological interpretation [19,51,53].

Registration also becomes more difficult in thick or physically expanded samples. Beyond global translational, rigid, or affine correction, 3D workflows may require local or deformable registration to account for tissue swelling, expansion anisotropy, section deformation, Z-axis drift, and local distortions between imaging cycles [12,19,28]. In multimodal settings, transcriptomic signals must be aligned with protein localization, morphology, or other spatial readouts, making cross-modal correspondence and joint inference significantly more difficult. In pathology-aware workflows, lesion geometry, FFPE section-derived image artifacts, pre-analytical variability, staining-induced deformation, and specimen-specific morphological distortions introduce additional alignment and interpretation burdens [14,34,35,36,70]. Cross-modal registration with H&E, immunostaining, or functional imaging is particularly challenging because these modalities differ in contrast mechanisms, spatial resolution, and deformation patterns, requiring deformable alignment and morphology-aware validation rather than simple image overlay [34,35,38].

A related infrastructure challenge is spatial data standardization. Imaging-based spatial transcriptomics produces heterogeneous data objects, including raw image volumes, decoded molecule tables, cell masks, cell-by-gene matrices, tissue annotations, coordinate transforms, and multimodal metadata. Community formats and frameworks such as open microscopy environment next-generation file formats and Zarr format (OME-NGFF/OME-Zarr) and SpatialData provide scalable ways to represent large bioimaging and spatial omics datasets, including chunked image storage, coordinate transformations, annotations, and multimodal data [41,42,43]. Large-scale atlas-building efforts such as the Human Cell Atlas, Human BioMolecular Atlas Program (HuBMAP), and the BRAIN Initiative Cell Census Network and Cell Atlas Network (BICCN/BICAN) are also generating public spatial and single-cell resources that can support method benchmarking, although task-specific ground truth for segmentation, decoding, and transcript assignment remains limited [81,84,85].

These settings also require more than local molecule recovery. Scalable visualization, atlas alignment, and three-dimensional reconstruction become part of the analytical problem itself rather than downstream presentation steps. The broader challenge is therefore no longer simply to detect molecules in space but to align molecular signals with anatomy, pathology, and multimodal context in ways that remain biologically coherent across scales and under uncertainty [34,35,38,70].

### 6.5. Training and Validation Requirements for Learning-Based Analysis

Deep learning-based models are increasingly used in imaging-based spatial transcriptomics for image restoration, spot detection, segmentation, transcript assignment, and reference-guided annotation, but their reliability depends strongly on the design of training and validation datasets [19,50,51,57,58]. Suitable training data should capture variation in tissue type, platform chemistry, imaging depth, signal density, staining quality, sample preparation, and pathology-associated artifacts, rather than relying only on visually clean fields of view [14,53,58]. Different tasks require different forms of supervision: restoration models often rely on paired low- and high-SNR images or simulated degradations, spot detectors require molecule-level annotations or reliable pseudo-labels, segmentation models require nuclear, membrane, or transcript-informed boundary evidence, and reference-mapping models require curated labels with documented uncertainty [17,19,50,57,58,71,74].

Public resources now provide useful starting points for model training and benchmarking. Large-scale atlas-building efforts such as the Human Cell Atlas, HuBMAP, and BICCN/BICAN have generated public single-cell, spatial, and multimodal resources that can support reference construction, label transfer, and cross-dataset validation [81,84,85]. Platform-specific spatial atlases, including whole-brain MERFISH and the CNS STARmap PLUS atlas, further provide large-scale examples for evaluating cell typing, spatial-domain inference, and atlas alignment [20,70,81]. For image segmentation, general and annotated tissue-imaging resources can also be found on the corresponding computational analysis platforms such as TissueNet/Mesmer and Cellpose to support robust model development [86,87,88].

However, existing public resources are not uniformly sufficient for all learning tasks in imaging-based spatial transcriptomics. Their adequacy depends on the intended test scenario: available datasets may be sufficient for pretraining, method development, or the stable evaluation of relatively generic image-segmentation tasks, especially when combined with modest task-specific fine-tuning, but they are often insufficient for robust deployment in new tissues, platforms, FFPE specimens, thick sections, or highly crowded RNA fields [53,58]. Current datasets are frequently fragmented across laboratories, platforms, staining protocols, and imaging systems, and few single experiments provide the scale and diversity needed for fully stable training and independent testing. In addition, complex tissues, rare cell types, disease specimens, and volumetric samples remain underrepresented. Many public datasets also lack matched membrane staining, 4′,6-diamidino-2-phenylindole (DAPI) staining, high-resolution H&E or immunofluorescence images, raw multicycle images, and expert-curated cell boundary or molecule-level annotations, all of which limit feature extraction and the construction of high-quality ground truth labels.

Validation should, therefore, extend beyond visual image quality or classification accuracy and include downstream molecular and biological criteria [19,49,53,58]. For restoration and spot detection, relevant criteria include molecule recovery, localization precision, false-positive and false-negative rates, decoding consistency, blank-code behavior when available, and reproducibility across fields of view or replicate samples [9,19,49,58]. For segmentation and transcript assignment, evaluation should include cell-by-gene matrix stability, marker-gene consistency, doublet or spillover behavior, and robustness across cell morphologies and tissue densities [17,64,65,67]. For reference mapping, transferred labels should be assessed together with confidence scores, marker expression, spatial localization, and unsupervised or de novo analyses to reduce bias toward predefined cell states [71,74]. Transparent reporting of training data, annotation strategies, held-out test sets, failure cases, uncertainty estimates, and cross-platform generalization is therefore essential for robust deployment [14,53,58].

## 7. Conclusions

Imaging-based spatial transcriptomics has progressed from low-plex single-molecule RNA imaging to a diverse methodological framework that spans cyclic hybridization, error-robust barcoding, in situ sequencing, volumetric profiling, multimodal readouts, and pathology-compatible workflows. Its distinctive value lies in preserving molecular identity and spatial context across multiple biological scales, from subcellular RNA localization to tissue architecture and atlas-scale organization.

However, imaging-based spatial transcriptomics is not defined by the assay chemistry alone. Its biological interpretability depends equally on computation: the field functions as an experiment–algorithm co-design framework in which image processing, decoding, segmentation, assignment, and spatial inference determine how imaging signals become biological knowledge.

The most important challenge ahead is no longer simply to measure more genes but to improve the fidelity and uncertainty awareness of spatial interpretation. In particular, future progress will depend on more reliable molecule-to-cell assignment, stronger integration across three-dimensional, multimodal, and pathology-aware data, and computational frameworks that preserve confidence information rather than collapsing uncertainty too early into hard calls. If these challenges can be addressed, imaging-based spatial transcriptomics will continue to move the field from descriptive molecular mapping toward a more mechanistic spatial systems biology approach.

## Figures and Tables

**Table 1:** Representative cell segmentation tools for imaging-based spatial transcriptomics.

Tool	Main Input	2D/3D Support	Nuclear or Membrane Staining Required	Algorithmic Strategy	Main Output	Code Availability
pciSeq	Transcript coordinates from in situ assays, nuclei/initial cell segmentation, scRNA-seq reference	Mainly 2D	Requires initial nuclei-guided or image-based segmentation	Probabilistic framework for transcript assignment and cell-type inference with boundary extension from nuclei	Transcript-to-cell assignment and probabilistic cell typing	Available
Baysor	Transcript coordinates; optional nuclear or cytoplasmic staining	2D and 3D	Optional	Probabilistic transcript-informed segmentation based on spatial proximity, morphology, and transcriptional composition	Cell boundaries and cell-by-gene matrix	Available
Proseg	Transcript coordinates from image-based spatial transcriptomics	Mainly 2D	Not strictly required	Unsupervised probabilistic modeling of transcript spatial distributions	Cell boundaries and transcript assignment	Available
CelloType	Multiplexed tissue images/image-based spatial omics data	Mainly 2D image-based	Requires image channels	Multitask learning for joint segmentation and classification	Instance masks and cell/object class labels	Available
Bering	Transcript coordinates and transcript colocalization graph; optional transferred embeddings	2D and 3D	Not strictly required	Graph-based learning for noise-aware segmentation and annotation	Cell segmentation and molecular annotation	Available

pciSeq relies on initial nuclei- or image-based segmentation and mainly performs probabilistic transcript assignment and cell typing, whereas Baysor, Proseg, CelloType, and Bering are designed for cell boundary inference or segmentation. “Available” indicates that public code has been reported by the authors. scRNA-seq refers to single-cell RNA-sequencing.

## Data Availability

No new data were created or analyzed in this study. Data sharing is not applicable to this article.

## Abbreviations

The following abbreviations are used in this manuscript:

ISH	in situ hybridization
ISS	in situ sequencing
RCA	rolling circle amplification
smFISH	single-molecule fluorescence in situ hybridization
MERFISH	multiplexed error-robust fluorescence in situ hybridization
seqFISH	sequential fluorescence in situ hybridization
osmFISH	ouroboros single-molecule fluorescence in situ hybridization
STARmap	spatially resolved transcript amplicon readout mapping
ExSeq	expansion sequencing
ExFISH	expansion fluorescence in situ hybridization
EASI-FISH	expansion-assisted iterative fluorescence in situ hybridization
FFPE	formalin-fixed paraffin-embedded
HybISS	hybridization-based in situ sequencing
RAEFISH	reverse-padlock amplicon-encoding fluorescence in situ hybridization
PRISM	profiling of RNA in situ through single-round imaging
FISH	fluorescence in situ hybridization
ISTDECO	in situ transcriptomics decoding by deconvolution
QC	quality control
H&E	hematoxylin and eosin
MOSAICA	multi omic single-scan assay with integrated combinatorial analysis
SNR	signal-to-noise ratio
CARE	content-aware image restoration
JSIT	joint sparse method for imaging transcriptomics
scRNA-seq	single-cell RNA-sequencing
SPLIT	spatial purification of layered intracellular transcripts
FICTURE	factor inference of cartographic transcriptome at ultra-high resolution
OME-NGFF/OME-Zarr	open microscopy environment next-generation file formats and Zarr format
HubMAP	Human BioMolecular Atlas Program
BICCN/BICAN	BRAIN Initiative Cell Census Network and Cell Atlas Network
DAPI	4′,6-diamidino-2-phenylindole

## References

[1] Mortazavi A., Williams B.A., McCue K., Schaeffer L., Wold B. (2008). Mapping and quantifying mammalian transcriptomes by RNA-Seq. Nat. Methods.

[2] Tang F., Barbacioru C., Wang Y., Nordman E., Lee C., Xu N., Wang X., Bodeau J., Tuch B.B., Siddiqui A. (2009). mRNA-Seq whole-transcriptome analysis of a single cell. Nat. Methods.

[3] Liu L., Chen A., Li Y., Mulder J., Heyn H., Xu X. (2024). Spatiotemporal omics for biology and medicine. Cell.

[4] Larsson L. (2021). Spatially resolved transcriptomics adds a new dimension to genomics. Nat. Methods.

[5] Chen K.H., Boettiger A.N., Moffitt J.R., Wang S., Zhuang X. (2015). Spatially resolved, highly multiplexed RNA profiling in single cells. Science.

[6] Ke R., Mignardi M., Pacureanu A., Svedlund J., Botling J., Wählby C., Nilsson M. (2013). In situ sequencing for RNA analysis in preserved tissue and cells. Nat. Methods.

[7] Ståhl P.L., Salmén F., Vickovic S., Lundmark A., Navarro J.F., Magnusson J., Giacomello S., Asp M., Westholm J.O., Huss M. (2016). Visualization and analysis of gene expression in tissue sections by spatial transcriptomics. Science.

[8] Williams C.G., Lee H.J., Asatsuma T., Vento-Tormo R., Haque A. (2022). An introduction to spatial transcriptomics for biomedical research. Genome Med..

[9] Eng C.H.L., Lawson M., Zhu Q., Dries R., Koulena N., Takei Y., Yun J., Cronin C., Karp C., Yuan G.-C. (2019). Transcriptome-scale super-resolved imaging in tissues by RNA seqFISH+. Nature.

[10] Codeluppi S., Borm L.E., Zeisel A., La Manno G., van Lunteren J.A., Svensson C.I., Linnarsson S. (2018). Spatial organization of the somatosensory cortex revealed by osmFISH. Nat. Methods.

[11] Wang X., Allen W.E., Wright M.A., Sylwestrak E.L., Samusik N., Vesuna S., Evans K., Liu C., Ramakrishnan C., Liu J. (2018). Three-dimensional intact-tissue sequencing of single-cell transcriptional states. Science.

[12] Alon S., Goodwin D.R., Sinha A., Wassie A.T., Chen F., Daugharthy E.R., Bando Y., Kajita A., Xue A.G., Marrett K. (2021). Expansion sequencing: Spatially precise in situ transcriptomics in intact biological systems. Science.

[13] Gyllborg D., Langseth C.M., Qian X., Choi E., Salas S.M., Hilscher M.M., Lein E.S., Nilsson M. (2020). Hybridization-based *in situ* sequencing (HybISS) for spatially resolved transcriptomics in human and mouse brain tissue. Nucleic Acids Res..

[14] Marco Salas S., Kuemmerle L.B., Mattsson-Langseth C., Tismeyer S., Avenel C., Hu T., Rehman H., Grillo M., Czarnewski P., Helgadottir S. (2025). Optimizing Xenium In Situ data utility by quality assessment and best-practice analysis workflows. Nat. Methods.

[15] Cheng Y., Dang S., Zhang Y., Chen Y., Yu R., Liu M., Jin S., Han A., Katz S., Wang S. (2025). Sequencing-free whole-genome spatial transcriptomics at single-molecule resolution. Cell.

[16] Chang T., Zhao S., Deng K., Liao Z., Tang M., Zhu Y., Han W., Yu C., Fan W., Jiang M. (2025). High-plex spatial RNA imaging in one round with conventional microscopes using color-intensity barcodes. Nat. Biotechnol..

[17] Petukhov V., Xu R.J., Soldatov R.A., Cadinu P., Khodosevich K., Moffitt J.R., Kharchenko P.V. (2022). Cell segmentation in imaging-based spatial transcriptomics. Nat. Biotechnol..

[18] Qian X., Harris K.D., Hauling T., Nicoloutsopoulos D., Muñoz-Manchado A.B., Skene N., Hjerling-Leffler J., Nilsson M. (2020). Probabilistic cell typing enables fine mapping of closely related cell types in situ. Nat. Methods.

[19] Fang R., Halpern A., Rahman M.M., Huang Z., Lei Z., Hell S.J., Dulac C., Zhuang X. (2024). Three-dimensional single-cell transcriptome imaging of thick tissues. eLife.

[20] Zhang M., Pan X., Jung W., Halpern A.R., Eichhorn S.W., Lei Z., Cohen L., Smith K.A., Tasic B., Yao Z. (2023). Molecularly defined and spatially resolved cell atlas of the whole mouse brain. Nature.

[21] Femino A.M., Fay F.S., Fogarty K., Singer R.H. (1998). Visualization of single RNA transcripts in situ. Science.

[22] Raj A., Van Den Bogaard P., Rifkin S.A., Van Oudenaarden A., Tyagi S. (2008). Imaging individual mRNA molecules using multiple singly labeled probes. Nat. Methods.

[23] Lubeck E., Coskun A.F., Zhiyentayev T., Ahmad M., Cai L. (2014). Single-cell in situ RNA profiling by sequential hybridization. Nat. Methods.

[24] Boström J., Zapaɫa M., Adameyko I. (2025). Boosting multiplexing capabilities for error-robust spatial transcriptomic methods using a set exchange approach. Sci. Adv..

[25] Wang G., Moffitt J.R., Zhuang X. (2018). Multiplexed imaging of high-density libraries of RNAs with MERFISH and expansion microscopy. Sci. Rep..

[26] Huang B., Babcock H., Zhuang X. (2010). Breaking the Diffraction Barrier: Super-Resolution Imaging of Cells. Cell.

[27] Chen F., Wassie A.T., Cote A.J., Sinha A., Alon S., Asano S., Daugharthy E.R., Chang J.-B., Marblestone A., Church G.M. (2016). Nanoscale imaging of RNA with expansion microscopy. Nat. Methods.

[28] Wang Y., Eddison M., Fleishman G., Weigert M., Xu S., Wang T., Rokicki K., Goina C., Henry F.E., Lemire A.L. (2021). EASI-FISH for thick tissue defines lateral hypothalamus spatio-molecular organization. Cell.

[29] Cui Y., Yang G., Goodwin D.R., O’fLanagan C.H., Sinha A., Zhang C., Kitko K.E., Shin T.W., Park D., Aparicio S. (2023). Expansion microscopy using a single anchor molecule for high-yield multiplexed imaging of proteins and RNAs. PLoS ONE.

[30] Sui X., Lo J.A., Luo S., He Y., Tang Z., Lin Z., Barabási D.L., Zhou Y., Wang W.X., Liu J. (2025). Scalable spatial single-cell transcriptomics and translatomics in 3D thick tissue blocks. Nat. Methods.

[31] Lee H., Salas S.M., Gyllborg D., Nilsson M. (2022). Direct RNA targeted in situ sequencing for transcriptomic profiling in tissue. Sci. Rep..

[32] Gataric M., Park J.S., Li T., Vaskivskyi V., Svedlund J., Strell C., Roberts K., Nilsson M., Yates L.R., Bayraktar O. (2021). PoSTcode: Probabilistic image-based spatial transcriptomics decoder. BioRxiv.

[33] Andersson A., Diego F., Hamprecht F.A., Wählby C. (2021). ISTDECO: In Situ Transcriptomics Decoding by Deconvolution. BioRxiv.

[34] Zeng H., Huang J., Zhou H., Meilandt W.J., Dejanovic B., Zhou Y., Bohlen C.J., Lee S.-H., Ren J., Liu A. (2023). Integrative in situ mapping of single-cell transcriptional states and tissue histopathology in a mouse model of Alzheimer’s disease. Nat. Neurosci..

[35] He S., Bhatt R., Brown C., Brown E.A., Buhr D.L., Chantranuvatana K., Danaher P., Dunaway D., Garrison R.G., Geiss G. (2022). High-plex imaging of RNA and proteins at subcellular resolution in fixed tissue by spatial molecular imaging. Nat. Biotechnol..

[36] Bass B.P., Engel K.B., Greytak S.R., Moore H.M. (2014). A Review of Preanalytical Factors Affecting Molecular, Protein, and Morphological Analysis of Formalin-Fixed, Paraffin-Embedded (FFPE) Tissue: How Well Do You Know Your FFPE Specimen?. Arch. Pathol. Lab. Med..

[37] Villacampa E.G., Larsson L., Mirzazadeh R., Kvastad L., Andersson A., Mollbrink A., Kokaraki G., Monteil V., Schultz N., Appelberg K.S. (2021). Genome-wide spatial expression profiling in formalin-fixed tissues. Cell Genom..

[38] Preibisch S., Innerberger M., León-Periñán D., Karaiskos N., Rajewsky N. (2025). Scalable image-based visualization and alignment of spatial transcriptomics datasets. Cell Syst..

[39] Kishi J.Y., Liu N., West E.R., Sheng K., Jordanides J.J., Serrata M., Cepko C.L., Saka S.K., Yin P. (2022). Light-Seq: Light-directed in situ barcoding of biomolecules in fixed cells and tissues for spatially indexed sequencing. Nat. Methods.

[40] Vu T., Vallmitjana A., Gu J., La K., Xu Q., Flores J., Zimak J., Shiu J., Hosohama L., Wu J. (2022). Spatial transcriptomics using combinatorial fluorescence spectral and lifetime encoding, imaging and analysis. Nat. Commun..

[41] Moore J., Allan C., Besson S., Burel J.-M., Diel E., Gault D., Kozlowski K., Lindner D., Linkert M., Manz T. (2021). OME-NGFF: A next-generation file format for expanding bioimaging data-access strategies. Nat. Methods.

[42] Marconato L., Palla G., Yamauchi K.A., Virshup I., Heidari E., Treis T., Vierdag W.-M., Toth M., Stockhaus S., Shrestha R.B. (2025). SpatialData: An open and universal data framework for spatial omics. Nat. Methods.

[43] Moore J., Basurto-Lozada D., Besson S., Bogovic J., Bragantini J., Brown E.M., Burel J.-M., Moreno X.C., de Medeiros G., Diel E.E. (2023). OME-Zarr: A cloud-optimized bioimaging file format with international community support. Histochem. Cell Biol..

[44] Martin N., Olsen P., Quon J., Campos J., Cuevas N.V., Nagra J., VanNess M., Maltzer Z., Gelfand E.C., Oyama A. (2025). MerQuaCo: A computational tool for quality control in image-based spatial transcriptomics. BioRxiv.

[45] Mao G., Yang Y., Luo Z., Lin C., Xie P. (2024). SpatialQC: Automated quality control for spatial transcriptome data. Bioinformatics.

[46] Sarder P., Nehorai A. (2006). Deconvolution methods for 3-D fluorescence microscopy images. IEEE Signal Process. Mag..

[47] Lohr D., Meyer L., Woelk L.M., Kovacevic D., Diercks B.P., Werner R., Diercks B.P. (2025). Deep Learning-Based Image Restoration and Super-Resolution for Fluorescence Microscopy: Overview and Resources. T Cell Activation.

[48] Perdigão L.M.A., Berger C., Yee N.B.Y., Darrow M.C., Basham M. (2024). RedLionfish—fast Richardson-Lucy Deconvolution package for efficient point spread function suppression in volumetric data. Wellcome Open Res..

[49] Wernersson E., Gelali E., Girelli G., Wang S., Castillo D., Langseth C.M., Verron Q., Nguyen H.Q., Chattoraj S., Casals A.M. (2024). Deconwolf enables high-performance deconvolution of widefield fluorescence microscopy images. Nat. Methods.

[50] Weigert M., Schmidt U., Boothe T., Müller A., Dibrov A., Jain A., Wilhelm B., Schmidt D., Broaddus C., Culley S. (2018). Content-aware image restoration: Pushing the limits of fluorescence microscopy. Nat. Methods.

[51] Bryan J.P., Binan L., McCann C., Eldar Y.C., Farhi S.L., Cleary B. (2023). Optimization-based decoding of Imaging Spatial Transcriptomics data. Bioinformatics.

[52] Chen S., Loper J., Chen X., Vaughan A., Zador A.M., Paninski L. (2021). BARcode DEmixing through Non-negative Spatial Regression (BarDensr). PLoS Comput. Biol..

[53] Belthangady C., Royer L.A. (2019). Applications, promises, and pitfalls of deep learning for fluorescence image reconstruction. Nat. Methods.

[54] Bahry E., Breimann L., Zouinkhi M., Epstein L., Kolyvanov K., Mamrak N., King B., Long X., Harrington K.I.S., Lionnet T. (2022). RS-FISH: Precise, interactive, fast, and scalable FISH spot detection. Nat. Methods.

[55] Mantes A.D., Herrera A., Khven I., Schlaeppi A., Kyriacou E., Tsissios G., Skoufa E., Santangeli L., Buglakova E., Durmus E.B. (2024). Spotiflow: Accurate and efficient spot detection for fluorescence microscopy with deep stereographic flow regression. BioRxiv.

[56] Laubscher E., Wang X., Razin N., Dougherty T., Xu R.J., Ombelets L., Pao E., Graf W., Moffitt J.R., Yue Y. (2024). Accurate single-molecule spot detection for image-based spatial transcriptomics with weakly supervised deep learning. Cell Syst..

[57] Eichenberger B.T., Zhan Y., Rempfler M., Giorgetti L., A Chao J. (2021). deepBlink: Threshold-independent detection and localization of diffraction-limited spots. Nucleic Acids Res..

[58] Xu W., Cai H., Zhang Q., Wang Z., Yang J., Wu X., Li C., Cui C., Liu C., He J. (2025). U-FISH: A fluorescent spot detector for imaging-based spatial-omics analysis and AI-assisted FISH diagnosis. Genome Biol..

[59] Sage D., Pham T.A., Babcock H., Lukes T., Pengo T., Chao J., Velmurugan R., Herbert A., Agrawal A., Colabrese S. (2019). Super-resolution fight club: Assessment of 2D and 3D single-molecule localization microscopy software. Nat. Methods.

[60] Xia C., Fan J., Emanuel G., Hao J., Zhuang X. (2019). Spatial transcriptome profiling by MERFISH reveals subcellular RNA compartmentalization and cell cycle-dependent gene expression. Proc. Natl. Acad. Sci. USA.

[61] Jones D.C., Elz A.E., Hadadianpour A., Ryu H., Glass D.R., Newell E.W. (2025). Cell simulation as cell segmentation. Nat. Methods.

[62] Pang M., Roy T.K., Wu X., Tan K. (2025). CelloType: A unified model for segmentation and classification of tissue images. Nat. Methods.

[63] Jin K., Zhang Z., Zhang K., Viggiani F., Callahan C., Tang J., Aronow B.J., Shu J. (2025). Bering: Joint cell segmentation and annotation for spatial transcriptomics with transferred graph embeddings. Nat. Commun..

[64] Wu L., Beechem J.M., Danaher P. (2025). Using transcripts to refine image based cell segmentation with FastReseg. Sci. Rep..

[65] Ergen C., Yosef N. (2025). ResolVI—addressing noise and bias in spatial transcriptomics. BioRxiv.

[66] Kwok A.W.C., Vannan A., Banovich N.E., Kropski J.A., Shim H., McCarthy D.J. (2025). Denoising image-based spatial transcriptomics data with DenoIST. Preprint. BioRxiv.

[67] Bilous M., Buszta D., Bac J., Kang S., Dong Y., Tissot S., Andre S., Gaveta M.A., Voize C., Peters S. (2026). Resolving sensitivity, specificity and signal contamination in Xenium spatial transcriptomics. Nat. Methods.

[68] Chen D.G., Ribas A., Campbell K.M. (2026). SpaceBender: Denoising Spatial Transcriptomics Data to Enhance Biological Signals. BioRxiv.

[69] Si Y., Lee C., Hwang Y., Yun J.H., Cheng W., Cho C.-S., Quiros M., Nusrat A., Zhang W., Jun G. (2024). FICTURE: Scalable segmentation-free analysis of submicron-resolution spatial transcriptomics. Nat. Methods.

[70] Shi H., He Y., Zhou Y., Huang J., Maher K., Wang B., Tang Z., Luo S., Tan P., Wu M. (2023). Spatial atlas of the mouse central nervous system at molecular resolution. Nature.

[71] Korsunsky I., Millard N., Fan J., Slowikowski K., Zhang F., Wei K., Baglaenko Y., Brenner M., Loh P.-R., Raychaudhuri S. (2019). Fast, sensitive and accurate integration of single-cell data with Harmony. Nat. Methods.

[72] Stuart T., Butler A., Hoffman P., Hafemeister C., Papalexi E., Mauck W.M., Hao Y., Stoeckius M., Smibert P., Satija R. (2019). Comprehensive Integration of Single-Cell Data. Cell.

[73] Chang J., Lu J., Liu Q., Xiang T., Zhang S., Yi Y., Li D., Liu T., Liu Z., Chen X. (2025). Single-cell multi-stage spatial evolutional map of esophageal carcinogenesis. Cancer Cell.

[74] Luecken M.D., Büttner M., Chaichoompu K., Danese A., Interlandi M., Mueller M.F., Strobl D.C., Zappia L., Dugas M., Colomé-Tatché M. (2022). Benchmarking atlas-level data integration in single-cell genomics. Nat. Methods.

[75] Benjamin K., Bhandari A., Kepple J.D., Qi R., Shang Z., Xing Y., An Y., Zhang N., Hou Y., Crockford T.L. (2024). Multiscale topology classifies cells in subcellular spatial transcriptomics. Nature.

[76] Zohora F.T., Paliwal D., Flores-Figueroa E., Li J., Gao T., Notta F., Schwartz G.W. (2025). CellNEST reveals cell–cell relay networks using attention mechanisms on spatial transcriptomics. Nat. Methods.

[77] Tejada-Lapuerta A., Schaar A.C., Gutgesell R., Palla G., Halle L., Minaeva M., Vornholz L., Dony L., Drummer F., Richter T. (2025). Nicheformer: A foundation model for single-cell and spatial omics. Nat. Methods.

[78] Zhang M., Eichhorn S.W., Zingg B., Yao Z., Cotter K., Zeng H., Dong H., Zhuang X. (2021). Spatially resolved cell atlas of the mouse primary motor cortex by MERFISH. Nature.

[79] Zhang Y., Watson B., Rattan A., Lee T., Chawla S., Geistlinger L., Guan Y., Lord F.B., Ma M., Miwa T. (2025). A spatial atlas of the complement system uncovers unique expression patterns in postnatal brain development in mice. Nat. Commun..

[80] Qian X., Coleman K., Jiang S., Kriz A.J., Marciano J.H., Luo C., Cai C., Manam M.D., Caglayan E., Lai A. (2025). Spatial transcriptomics reveals human cortical layer and area specification. Nature.

[81] Yao Z., Van Velthoven C.T.J., Kunst M., Zhang M., McMillen D., Lee C., Jung W., Goldy J., Abdelhak A., Aitken M. (2023). A high-resolution transcriptomic and spatial atlas of cell types in the whole mouse brain. Nature.

[82] Morad G., Damania A.V., Melendez B., Singh B.B., Veguilla F.J., Soto R.A., Hoballah Y.M., Sahasrabhojane P.V., Wong M.C., Ahmed M.M. (2025). Microbial signals in primary and metastatic brain tumors. Nat. Med..

[83] Sun Y., Zhang M., Zhao Y., Yan Y., Wang L., Liu X., Xia S., Wang B., Zhang X., Wang Y. (2025). Spatial transcriptomics reveals macrophage domestication by epithelial cells promotes immunotherapy resistance in small cell lung cancer. NPJ Precis. Oncol..

[84] Regev A., Teichmann S.A., Lander E.S., Amit I., Benoist C., Birney E., Bodenmiller B., Campbell P., Carninci P., Clatworthy M. (2017). Science forum: The Human Cell Atlas. eLife.

[85] Snyder M.P., HuBMAP Consortium, Writing Group (2019). The human body at cellular resolution: The NIH Human Biomolecular Atlas Program. Nature.

[86] Stringer C., Wang T., Michaelos M., Pachitariu M. (2021). Cellpose: A generalist algorithm for cellular segmentation. Nat. Methods.

[87] Pachitariu M., Stringer C. (2022). Cellpose 2.0: How to train your own model. Nat. Methods.

[88] Greenwald N.F., Miller G., Moen E., Kong A., Kagel A., Dougherty T., Fullaway C.C., McIntosh B.J., Leow K.X., Schwartz M.S. (2022). Whole-cell segmentation of tissue images with human-level performance using large-scale data annotation and deep learning. Nat. Biotechnol..

